# Einschränkungen der Mobilität von älteren Verkehrsteilnehmern durch Medikamente, Alkohol und Cannabis

**DOI:** 10.1007/s00103-024-03913-6

**Published:** 2024-06-24

**Authors:** Theresa Bödefeld, Benno Hartung

**Affiliations:** 1 Institut für Rechtsmedizin, Forensische Toxikologie, Universitätsklinikum Essen, Hufelandstraße 55, 45122 Essen, Deutschland; 2 Institut für Rechtsmedizin, Universitätsklinikum Essen, Essen, Deutschland

**Keywords:** Fahrsicherheit, Fahreignung, Benzodiazepine, Opioide, Antidepressiva, Driving safety, Fitness to drive, Benzodiazepines, Opioids, Antidepressants

## Abstract

Viele Grunderkrankungen gehen mit Symptomen einher, die die Fähigkeit, komplexe Aufgaben des Alltags zu lösen, beeinträchtigen können. Hierzu gehört auch die aktive Teilnahme am Straßenverkehr. Das Ziel von Arzneimitteltherapien ist es, sofern keine Heilung möglich ist, die Symptome soweit zu lindern, dass der Patient im Alltag so wenig Einschränkungen wie möglich erfährt. Jedoch haben etwa 20 % der ca. 100.000 in Deutschland zugelassenen Arzneimittel verkehrsrelevante Nebenwirkungen, die die Fahrsicherheit ihrerseits ebenfalls beeinträchtigen können.

So wird davon ausgegangen, dass an jedem 4. Verkehrsunfall die Wirkung eines Arzneimittels zumindest mitursächlich ist und jeder 10. Verkehrstote Psychopharmaka vor Fahrtantritt eingenommen hat. Neben Alkohol und Drogen stehen insbesondere Medikamente aus der Gruppe der Benzodiazepine, Opioide und Antidepressiva im Verdacht, die Fahrsicherheit zu beeinträchtigen. Die Effekte dieser Substanzen auf junge Menschen sind vielfach beschrieben. In dieser Übersichtsarbeit sollen speziell die verkehrsrelevanten (Neben‑)Wirkungen verschiedener Wirkstoffklassen auf betagte Personen ab 65 Jahren dargestellt werden.

Insbesondere Ältere müssen häufig verschiedene Medikamente einnehmen, die aufgrund von Grunderkrankungen anders metabolisiert werden als bei Jüngeren und darüber hinaus interagieren können. Es kann festgestellt werden, dass (1) ältere Personen häufig sensibler auf Substanzen reagieren, (2) nicht alle Vertreter einer Wirkstoffklasse den gleichen Effekt auf die Fahrsicherheit haben und (3) eine pauschale Beurteilung nicht möglich ist, sondern die Wirkungen von weiteren Faktoren, wie zum Beispiel Grunderkrankungen, Therapieregime und Einnahmezeit der Medikation, abhängen.

## Abstract (en)

Many diseases are accompanied by symptoms that can impair the ability to perform complex everyday tasks, such as active participation in road traffic. If a cure is not possible, the aim of drug therapy is to alleviate the symptoms to such an extent that the patient no longer has any restrictions in everyday life. However, around 20% of the approximately 100,000 medicines licensed in Germany have traffic-relevant side effects that can also lead to driving impairment.

It is assumed that the effect of a drug is at least partially responsible for one in four traffic accidents and that one in ten victims of fatal road accidents has taken psychotropic drugs before driving. In addition to alcohol and drugs, medications from the benzodiazepine, opioid, and antidepressant groups are suspected of impairing driving safety in particular. The effects of these substances on young people have been described many times, but this review deals specifically with the traffic-relevant (side) effects of various classes of drugs on elderly people (aged 65 and over).

Older people in particular often have to take different medications, which are metabolized differently compared to younger people due to underlying diseases and can also interact with each other. It was found that (1) older people often react more sensitively to substances, (2) not all representatives of a drug class have the same effect on driving safety, and (3) a general assessment of a drug’s safety is not possible, since the effects also depend on other factors such as underlying diseases, treatment regimen, and the time of day the medication is taken.

## Einleitung

Auto zu fahren bedeutet für die meisten Menschen Unabhängigkeit und gerade im Alter, wenn Einschränkungen nicht plötzlich, sondern graduell zunehmen, fällt es oft schwer, die eigene Fahrunsicherheit oder gar fehlende Fahreignung zu erkennen und auf das Auto zu verzichten.

Die Gründe für eine verminderte Fahrsicherheit bzw. fehlende Fahreignung von betagten Personen sind vielfältig. So können zum Beispiel die Symptome vieler Krankheiten bereits die Fahrsicherheit/Fahreignung einschränken oder sogar aufheben.

Häufig kann die Fahreignung nur durch die Einnahme von Medikamenten wiederhergestellt werden: Zum Beispiel dürfen Epilepsie-Patienten erst dann wieder Auto fahren, wenn sie mindestens ein Jahr lang anfallsfrei waren, dies gelingt jedoch häufig nur durch die Einnahme von Antiepileptika. Es konnte auch gezeigt werden, dass die Einnahme von Calciumkanalblockern und Vasodilatatoren, wie sie zur Behandlung verschiedener Herzerkrankungen verschrieben werden, mit einem verminderten Risiko für Verkehrsunfälle assoziiert ist [1].

Jedoch führt die Einnahme von Medikamenten nicht immer und insbesondere nicht zu Beginn der Behandlung dazu, dass die Fahrsicherheit/Fahreignung wiederhergestellt wird, sondern neben den Grunderkrankungen kann die Arzneimitteleinnahme die Fahrsicherheit/Fahreignung beeinträchtigen. An der Entstehung von etwa jedem 4. Verkehrsunfall sind Wirkungen bzw. Nebenwirkungen eines Arzneimittels beteiligt und man geht davon aus, dass etwa jeder 10. Verkehrstote unter dem Einfluss von Psychopharmaka stand [2]. Toxikologische Untersuchungen von Blutproben aufgrund von Fahrauffälligkeiten im Straßenverkehr sind bei Senioren zwar deutlich unterrepräsentiert, jedoch werden in diesen Fällen die verkehrsmedizinisch hoch relevanten Benzodiazepine überproportional häufig nachgewiesen [3].

Auch wenn viele Patienten nicht wissen, dass neben Drogen und Alkohol auch Arzneimittel die sichere Teilnahme am Straßenverkehr beeinträchtigen können, sind die Effekte verschiedener Arzneistoffklassen auf psychomotorische und kognitive Leistungen bekannt. In diesem Zusammenhang ist es wichtig zu wissen, dass das sogenannte Arzneimittel-Privileg nach § 24a Straßenverkehrsgesetz (StVG), wonach keine Ordnungswidrigkeit vorliegt, „wenn die Substanz aus der bestimmungsgemäßen Einnahme eines für einen konkreten Krankheitsfall verschriebenen Arzneimittels herrührt“, im Strafrecht (§§ 315c und 316 StGB) nicht gilt. Demnach ist es strafbar, wenn jemand nach Substanzkonsum „nicht in der Lage ist, das Fahrzeug sicher zu führen“ (§ 316 StGB), oder „nicht in der Lage ist, das Fahrzeug sicher zu führen und dadurch Leib oder Leben eines anderen oder fremde Sachen von bedeutendem Wert gefährdet“ (§ 315c StGB).

Bereits 1964 veröffentlichte die Bundesärztekammer ein „Merkblatt über die Einschränkung der Verkehrstüchtigkeit durch Arzneimittel“, in dem auf die Pflicht des Herstellers hingewiesen wird, Arzneimittel auf Wirkungen untersuchen zu lassen, die die Verkehrssicherheit einschränken können, und in der Arztinformation und Packungsbeilage auf mögliche Gefahren in Bezug auf die Verkehrsteilnahme hinzuweisen [4].

Von den mittlerweile etwa 100.000 in Deutschland zugelassenen Arzneimitteln sind ca. 20 % verkehrsmedizinisch relevant, da sie Reaktionszeit, Aufmerksamkeit, visuelle Funktionen, Motorik sowie kognitive Leistungen beeinträchtigen können [5]. Eine retrospektive Kohortenstudie zeigte auf, dass das relative Risiko (RR) für einen Verkehrsunfall mit Verletzungsfolge unter der Einnahme von psychoaktiven Medikamenten erhöht ist (RR 1,5; 95 %-Konfidenzintervall (KI): 1,2–1,9; [6]).

Auf die gesetzlich Krankenversicherten ab 65 Jahren, die ca. 22 % der Gesamtpopulation ausmachen, entfallen 55,9 % des DDD-Volumens („defined daily dose“). Im Durchschnitt werden Personen ab 65 Jahren somit täglich mit 4,3 Tagesdosen verschiedener Arzneimittel behandelt [7].

Eine Möglichkeit zur generellen Einschätzung des Gefahrenpotenzials verschiedener Medikamente bietet die Kategorisierung des ICADTS (International Council on Alcohol, Drugs and Traffic Safety). Diese bezieht sowohl Daten aus dem pharmakologischen Wirkprofil als auch Ergebnisse aus standardisierten Fahrten mit ein [5]. Eine weitere Einschätzung der Nebenwirkungen, die speziell bei betagten Personen auftreten können, bietet die *Priscus*-Liste [8], welche zudem alternative, besser geeignete Präparate oder Dosierungen vorschlägt und des Weiteren auch Ratschläge bezüglich einer möglichen Überprüfung einer sicheren Arzneimitteltherapie bietet.

Die Gefährdung des Straßenverkehrs nimmt erheblich zu, wenn neben Medikamenten (insbesondere Psychopharmaka) zusätzlich Alkohol oder andere Drogen konsumiert werden. Gemäß DRUID-Studie erhöht sich die Odds Ratio (OR) bei gleichzeitiger Einnahme von Drogen/Medikamenten und Alkohol auf 20–200 [9].

Im Rahmen dieser Übersichtsarbeit werden mögliche verkehrsrelevante Auswirkungen einer medikamentösen Therapie und anderer zentralwirksamer Substanzen speziell bei Personen ab 65 Jahren beleuchtet mit dem Ziel, Substanzen/Substanzgruppen zu identifizieren, welche die Fahrsicherheit von diesen Menschen beeinträchtigen können, und ggf. risikoärmere Alternativpräparate zu finden.

## Übersicht der Wirkstoffklassen und deren (Neben‑)Wirkungen

### Arzneimittel

#### Schlaf‑/Beruhigungsmittel: Benzodiazepine und Z-Substanzen

Benzodiazepine wirken vorwiegend schlafanstoßend, antikonvulsiv und muskelrelaxierend. Als verkehrsmedizinische Nebenwirkungen können unter anderem Einschränkungen der kognitiven Fähigkeiten, Schwindel, Sedierung, Verwirrtheitszustände sowie Muskelschwäche auftreten. Damit einhergehende (Fahr‑)Auffälligkeiten sind Schlangenlinienfahren, Sprachstörungen und Gangunsicherheiten [10]. Insbesondere bei älteren Menschen können auch paradoxe Erregungs- und Verwirrtheitszustände auftreten, die u. a. mit Halluzinationen einhergehen. Das relative Risiko, im Rahmen eines Verkehrsunfalls schwer oder tödlich verletzt zu werden, wird durch Benzodiazepine und Z‑Substanzen um das 2‑ bis 10-Fache erhöht [9]. Man unterscheidet Benzodiazepine zum einen nach ihrer Halbwertszeit in kurz- (< 8 h), mittel- (8–24 h) und langwirksame (> 24 h) Benzodiazepine und zum anderen nach ihrer Indikation als Anxiolytika oder Hypnotika.

Epidemiologische Studien haben gezeigt, dass Benzodiazepine eine große Rolle im Rahmen von Verkehrsunfällen spielen [11, 12]. Dabei handelt es sich jedoch nicht nur um Effekte, die unmittelbar oder wenige Stunden nach der Einnahme auftreten. Verlängerte Wirkungen (Residualwirkungen, „Hangover-Effekte“), wie Einschränkungen der psychomotorischen Leistungsfähigkeit, der visuellen Wahrnehmung, der Aufmerksamkeit, der Gedächtnisleistung und der Informationsverarbeitung, können die Fahrsicherheit auch noch am nächsten Tag beeinträchtigen [13]. Diese Effekte sind sowohl dosisabhängig als auch abhängig von der Halbwertszeit und der Darreichungsform/Herstellung (Galenik) des Arzneimittels. Insbesondere bei der Einnahme von langwirksamen Benzodiazepinen treten Residualeffekte regelhaft auf. Leufkens und Vermeeren haben im Rahmen ihrer Untersuchungen beobachtet, dass sich die Residualeffekte nach der Einnahme von Hypnotika bei älteren Patienten nicht signifikant von denen bei jüngeren unterscheiden [14], jedoch gibt es auch Untersuchungen, die eine höhere Sensitivität von Älteren gegenüber Hypnotika beschreiben [15]. Insbesondere hoch dosiertes Flurazepam scheint für betagte Personen deutlich schlechter verträglich zu sein als für weniger betagte. Bei Dosierungen von 30 mg/Tag und mehr konnten bei 39 % der über 70-Jährigen unerwünschte Nebenwirkungen wie eine Dämpfung des zentralen Nervensystems beobachtet werden. Niedrigere Dosierungen scheinen für ältere Personen hingegen besser verträglich zu sein, so wurden bei Dosierungen von unter 15 mg/Tag nur bei 2 % der über 70-Jährigen Nebenwirkungen beschrieben [16]. Das Risiko für einen Verkehrsunfall unter Benzodiazepintherapie wird bei betagten Personen teilweise als höher angesehen als bei Personen unter 65 Jahren [6, 17, 18]. Andere Arbeiten legen dies nicht nahe [11, 12, 19] oder sehen hierin eher ein altersunabhängiges Problem [20].

Die als Schlafmittel (Hypnotika) verordneten Benzodiazepine werden häufig beim Zubettgehen eingenommen. In Abhängigkeit von ihrer Halbwertszeit können sie bei Fahrtantritt am nächsten Tag bereits wieder soweit abgebaut sein, dass sie keine verkehrsrelevanten Nebenwirkungen mehr zeigen. So konnte vor allem für kurz- bis mittellangwirksame Benzodiazepine, wie z. B. Temazepam [14] und Midazolam [21], gezeigt werden, dass das Unfallrisiko am Tag nach der Einnahme nicht signifikant erhöht ist [11, 14, 17].

Benzodiazepine, die bei Angstzuständen eingesetzt werden (Anxiolytika) werden häufig tagsüber angewendet. Nebenwirkungen machen sich demzufolge bereits bei Substanzen mit kurzer Halbwertszeit bemerkbar und können das Unfallrisiko im Vergleich zu Hypnotika dosisabhängig erhöhen [11]. Die Einnahme von Alprazolam als Anxiolytikum führte im Rahmen einer Placebo-kontrollierten Studie vermehrt zu Verkehrseinbußen, die sich zum Beispiel in Störungen der Aufmerksamkeit und im Schlangenlinienfahren (gemessen als Standardabweichung von der lateralen Position – SDLP) zeigten [22].

Des Weiteren wurde in verschiedenen Studien untersucht, ob das Unfallrisiko unter anhaltender Benzodiazepintherapie weiterhin erhöht bleibt. Nach der Einnahme von Diazepam, welches aufgrund seiner Metabolisierung in ebenfalls pharmakologisch aktive Metaboliten eine sehr lange Halbwertszeit aufweist, zeigten sich auch noch nach 1–4 Wochen Behandlungsdauer Nebenwirkungen, die die Fahrsicherheit signifikant einschränken können [19, 23–25], wobei das Risiko innerhalb der ersten Behandlungswoche am höchsten war [12]. Für Oxazepam wurde ein solch erhöhtes Unfallrisiko nach dieser Behandlungsdauer nicht mehr detektiert [19]. Der Langzeitgebrauch von Benzodiazepinen scheint in geringerem Ausmaß zu verkehrsrelevanten Nebenwirkungen zu führen [17, 26].

Eine Weiterentwicklung der Benzodiazepine stellen die Z‑Substanzen (Benzodiazepin-Analoga, wie Zopiclon, Zolpidem und Zaleplon) dar. Diese binden trotz ihrer andersartigen Struktur im Bereich der Benzodiazepin-Bindungsstelle an den GABA_A_-Rezeptor, dennoch sollen durch diese Z‑Substanzen Schlafphasen weniger beeinträchtigt werden und die Gefahr der Abhängigkeit geringer sein als unter Benzodiazepintherapie [27]. Im Rahmen einer Fall-Kontroll-Studie konnten Barbone et al. zeigen, dass auch bei den neueren Benzodiazepin-Analoga verkehrsrelevante Nebenwirkungen und Residualeffekte auftreten können [11]. Das Unfallrisiko nach der Einnahme von Zopiclon ist etwa 4‑fach erhöht ([11, 13]; OR 4,00; 95 %-KI: 1,31–12,2; [11]). Eine Beeinträchtigung der Fahrsicherheit unter Zopiclon-Einnahme konnte auch noch 11 h nach der Einnahme beobachtet werden [14]. Interessanterweise fühlten sich die Studienteilnehmer unter der Einnahme von Zopiclon nicht deutlich unaufmerksamer als unter Placebo. Die Fahrsicherheit bezog sich in diesen Untersuchungen sowohl auf die Fähigkeit, die Spur zu halten, als auch auf Gedächtnisleistung und Aufmerksamkeit [13]. Gustavsen et al. kamen in ihrer Kohortenstudie auf ein 2‑fach erhöhtes Risiko sowohl bei Zopiclon als auch bei Zolpidem [25]. Für Zaleplon konnten aufgrund der extrem kurzen Halbwertszeit von etwa einer Stunde und dem Abbau in inaktive Metaboliten bereits 3 h nach der Einnahme keine negativen Auswirkungen auf das Gedächtnis und die psychomotorische Leistungsfähigkeit mehr festgestellt werden [13].

#### Starke Schmerz- und Hustenmittel: Opioide

Opioide werden in der Regel bei Erkrankungen, die mit starken Schmerzen einhergehen, sowie als Hustenmittel (überwiegend Codein) verordnet. Aufgrund ihrer Fähigkeit, ins zentrale Nervensystem zu gelangen, können neben der schmerzstillenden Wirkung vor allem auch Sedierung, Schwindel, Blutdrucksenkung, Verwirrtheitszustände sowie Sehstörungen durch eine Engstellung der Pupillen auftreten. Das relative Risiko, im Rahmen eines Verkehrsunfalls schwer oder tödlich verletzt zu werden, wird durch Opioide um das 2‑ bis 10-Fache erhöht [9].

Opioide werden aufgrund ihrer Wirkungen häufig auch missbräuchlich verwendet, was nicht nur die Fahrsicherheit beeinflussen kann, sondern auch eine Fahreignung ausschließt. Eine Unterscheidung zwischen missbräuchlicher Einnahme von Opioiden und ärztlich verordneter Medikation ist häufig nur mithilfe der Krankenunterlagen möglich, da selbst Heroin in Ausnahmefällen als Medikament eingesetzt wird und alle Opioide, insbesondere Fentanyl oder Oxycodon, auch missbräuchlich verwendet werden.

Die Wirkung von Opioid-Analgetika auf die Fahrsicherheit von betagten Personen wurde bereits im Rahmen von verschiedenen Studien weitgehend untersucht, je nach Studiendesign und Betrachtungsweise sind die Ergebnisse jedoch recht unterschiedlich. So haben Leveille et al. im Rahmen ihrer Untersuchungen ein 2‑fach erhöhtes Risiko für Verkehrsunfälle mit Verletzungsfolge bei älteren Personen unter Opioid-Einnahme beobachtet [18]. Ray et al. konnten hingegen keine Assoziation zwischen Opioiden und Verkehrsunfällen mit Verletzungsfolge finden [6]. Leveille et al. vermuten den Unterschied in der Einnahme von Codein als Hustenmittel, welches in den Studien von Ray et al. ausgeschlossen, in ihre eigene Studie aber miteingeschlossen worden ist. Aufgrund der verminderten Nierenleistung bei betagten Personen können diese jedoch sensitiver auf die sedierenden Effekte von Codein reagieren, sodass die verschiedenen Ergebnisse der beiden Studien für das Unfallrisiko laut Leveille et al. darauf zurückzuführen sein können [18].

Insbesondere bei neu angesetzter Therapie bzw. Einzeldosen von Opioiden wurden bei freiwilligen Versuchspersonen negative Auswirkungen auf die Konzentrationsfähigkeit, Aufmerksamkeitsbelastung, Reaktionszeit und Muskelkoordination festgestellt, die für eine sichere Verkehrsteilnahme unabdingbar sind. Auch bei 4‑wöchiger oraler Opioid-Therapie wurden weiterhin dosisabhängige, verkehrsrelevante Nebenwirkungen beobachtet, die bei älteren Patienten und bei Malignompatienten signifikant stärker ausgeprägt waren als bei jüngeren Personen ohne Malignomschmerzen. Im optischen und akustischen Reaktionstest haben Studienteilnehmer der Opioid-Gruppe hochsignifikant langsamer reagiert als die Kontrollgruppe und auch hier bestand ein signifikanter Zusammenhang mit steigendem Lebensalter. Synergistische Effekte durch die zusätzliche Einnahme von Antidepressiva konnten jedoch nicht ausgeschlossen werden [28].

Eine Opioid-Therapie führt nicht zwangsläufig zu Nebenwirkungen, die eine aktive Teilnahme am Straßenverkehr ausschließen, sondern muss je nach Einzelfall individuell betrachtet werden. Eine Verkehrsteilnahme sollte nur während der Einstellungsphase auf Opioide, bei Dosiskorrekturen (Erhöhung, Reduktion), bei Wechsel des Opioids sowie bei schlechtem Allgemeinzustand generell untersagt werden [29]. Bei langfristiger Opioid-Einnahme stellt sich jedoch eine Gewöhnung sowohl in Hinblick auf die Hauptwirkung als auch auf einige Nebenwirkungen ein, sodass bei einem stabilen Therapieverlauf und gutem Allgemeinzustand die Teilnahme am Straßenverkehr wieder möglich sein kann, sofern der Patient sich vor Fahrtantritt im Hinblick auf die Fahrsicherheit selbstkritisch prüft [29].

Da die Pupillen jedoch auch bei stabil eingestellter Therapie aufgrund der Opioide weiterhin eng gestellt sein können und hier keine Gewöhnung auftritt, besteht aufgrund der Störung der Dunkeladaptation insbesondere bei Nachtfahrten dauerhaft eine erhöhte Unfallgefahr.

#### Antidepressiva/Psychopharmaka

Antidepressiva/Psychopharmaka werden nicht nur bei Depressionen eingesetzt, sondern können auch eine Komponente der Schmerztherapie darstellen oder als Schlaf‑/Beruhigungsmittel eingesetzt werden. Aufgrund der Vielzahl an unterschiedlichen Wirkstoffklassen innerhalb dieser Substanzgruppe können auch mögliche Nebenwirkungen vielfältig sein. Potenziell verkehrsmedizinisch relevante Nebenwirkungen sind vor allem Sedierung, Schwindel, Verwirrtheitszustände, Seh- und Bewegungsstörungen, extrapyramidal-motorische Störungen sowie Durchblutungsstörungen des Gehirns. Im Rahmen einer pharmakoepidemiologischen Studie aus den Niederlanden konnte festgestellt werden, dass die Einnahme von modernen Antidepressiva das Risiko eines Verkehrsunfalls nahezu verdoppelt (OR 1,76; 95 %-KI: 1,4–2,2; [9]).

Insbesondere bei den trizyklischen Antidepressiva, die im Vergleich zu den selektiven Serotonin-Wiederaufnahmehemmern stärker sedierend wirken können, kann die Fahrsicherheit nach der Einnahme beeinträchtigt sein [5].

Depressionen gehen jedoch generell mit einer verlangsamten Reaktionszeit [30] und mit verminderter Aufmerksamkeit [31] einher, sodass behandelte Personen mit Depressionen in Untersuchungen eine bessere Fahrperformance gezeigt haben als unbehandelte [32].

Barbone et al. konnten in Bezug auf trizyklische Antidepressiva (OR 0,93; 95 %-KI: 0,72–1,21) und selektive Serotonin-Wiederaufnahmehemmer (OR 0,85; 95 %-KI: 0,55–1,33) keinen Effekt auf das Unfallrisiko beobachten [11].

2 epidemiologische Studien [6, 18], die den Effekt von Antidepressiva bei Personen über 60 Jahren untersucht haben, konnten ein erhöhtes Unfallrisiko nach der Einnahme von trizyklischen Antidepressiva beobachten, welches dosisabhängig ist (OR 2,3; 95 %-KI: 1,1–4,8; [18] bzw. RR 2,2; 95 %-KI: 1,3–3,5; [6]). Nach der Einnahme von Amitriptylin in Dosierungen von 125 mg/Tag oder mehr war das Unfallrisiko sogar noch deutlicher erhöht (RR 5,5; 95 %-KI: 2,6–11,6; [6]). Auch Bramness et al. konnten nach der Einnahme von Antidepressiva einen leichten Anstieg des Unfallrisikos sehen, wobei unklar blieb, ob das Risiko durch die Grunderkrankung oder das Medikament erhöht wird [33]. Die beiden selektiven Serotonin-Wiederaufnahmehemmer Paroxetin [34] und Fluoxetin [35] scheinen im Gegensatz zu den trizyklischen Antidepressiva nur einen geringen Einfluss auf die Fahrsicherheit zu haben.

### Alkohol und andere Drogen

#### Alkohol

Auch wenn die Assoziation zwischen Alkoholkonsum und Verletzungen bereits im alten Ägypten bekannt war [36] und auch schon erste Studien zur Kausalität von Alkoholkonsum und Verkehrsunfällen 1938 durch Holcomb [37] durchgeführt worden sind, so ist jedoch spätestens seit der Grand-Rapids-Studie in den Jahren 1962 und 1963 die Beteiligung von Alkohol an Verkehrsunfällen Teil des Allgemeinwissens [38]. Das relative Risiko, im Rahmen eines Verkehrsunfalls schwer oder tödlich verletzt zu werden, ist abhängig von der Blutalkoholkonzentration (BAK). Bei einer BAK von ≤ 0,5–< 0,8 ist das relative Risiko 2‑ bis 10-fach erhöht, bei einer BAK von ≤ 0,8–< 1,2 g/l 5‑ bis 30-fach und bei einer BAK von ≥ 1,2 g/l sogar 20- bis 200-fach [9].

Im Kontrast zu den gewünschten, als sozial geltenden Wirkungen des Alkohols wie Aufgeschlossenheit, Redseligkeit etc. stehen die verkehrsrelevanten Nebenwirkungen, die in Abhängigkeit von dem gewählten Verkehrsmittel zu selbst- und/oder fremdgefährdendem Verhalten führen. Kognitive (Aufmerksamkeit, Konzentration und Reaktion) wie auch psychomotorische Defizite (Koordinationsstörungen, Geh‑/Standunsicherheiten, verwaschene Aussprache) kommen regelmäßig vor.

Die psychophysischen Leistungseinbußen korrelieren bei Alkohol – im Gegensatz zu den meisten anderen Drogen – relativ stabil mit der Blutalkoholkonzentration und sind in der Anflutungsphase deutlich stärker ausgeprägt als bei gleicher Konzentration in der Eliminationsphase, weswegen insbesondere nach Sturztrunkphasen überproportional stärkere Ausfallerscheinungen zu erwarten sind [39].

Zudem kann es bei Personen, die neben dem Alkohol auch noch Medikamente zu sich nehmen, schon bei geringem Alkoholkonsum zu starken Wechselwirkungen zwischen dem Alkohol und dem Arzneistoff kommen [20]. Insbesondere Wirkstoffe mit zentraldämpfender Wirkung wie Opioide und Schlafmittel können additive Effekte mit der sedierenden Komponente des Alkohols aufweisen.

#### Cannabis

Als Cannabinoide bezeichnet man die für die Hanfpflanze (*Cannabis sativa var. indica*) charakteristischen Terpenphenolverbindungen, von denen das Tetrahydrocannabinol (THC) sowie seine Metaboliten 11-Hydroxy-THC (psychotrop) und die THC-Carbonsäure (inaktiv) von forensischem Interesse sind.

Cannabis kann sowohl ärztlich verordnet als auch als illegale Rauschdroge konsumiert werden. Eine Unterscheidung zwischen medizinischem Cannabis und der Rauschdroge ist analytisch nicht möglich und auch die (Neben‑)Wirkungen verschiedener Sorten sind vergleichbar.

Für einen typischen Cannabisrausch wurden Wirkungen wie Euphorie, Antriebsminderung, Konzentrations- und Wahrnehmungsstörungen, Denkstörungen sowie Änderungen des Zeiterlebens beschrieben. Typische Fahrfehler nach dem Konsum von Cannabis sind wechselnde Fahrgeschwindigkeiten sowie Abkommen von der Fahrspur mit anschließender Lenkkorrektur [39]. Das relative Risiko, im Rahmen eines Verkehrsunfalls schwer oder tödlich verletzt zu werden, wird durch den Konsum von Cannabis 1‑ bis 3‑fach erhöht [9].

Im Rahmen einer aktuellen Untersuchung zum Fahrverhalten in einem Fahrsimulator unter Cannabiseinfluss von 31 Probanden im Alter zwischen 65 und 79 Jahren konnten kurz nach dem Cannabiskonsum verkehrsmedizinisch relevante Auffälligkeiten (SDLP) bei gleichzeitig reduzierter Durchschnittsgeschwindigkeit festgestellt werden [40], sodass hier Analogien zu jüngeren Personengruppen zu erkennen sind.

## Diskussion

Medikamente können zum einen die Fahrsicherheit/Fahreignung, welche aufgrund von einer oder mehrerer Grunderkrankungen aufgehoben war, wiederherstellen, zum anderen jedoch auch selbst die Fahrsicherheit stark beeinträchtigen. Die medikamentös verursachten Beeinträchtigungen finden sich vor allem während der Einstellungsphase.

Gerade bei multimorbiden Patienten wird häufig eine Vielzahl von Präparaten verordnet (sog. Polypharmazie), die nicht nur für sich allein genommen schwerwiegende Nebenwirkungen haben, die die Fahrsicherheit beeinträchtigen können, sondern zusätzlich interagieren. Darüber hinaus können einige Präparate auch speziell bei betagten Menschen zum Beispiel aufgrund von Grunderkrankungen wie Leber‑/Nierenfunktionsstörungen Nebenwirkungen aufweisen, die bei jüngeren nicht auftreten. Insbesondere bei neuer Medikation oder Dosisanpassung (Erhöhung/Reduktion), aber auch nach dem Absetzen von Medikamenten hat der Fahrzeuglenker vor Fahrtantritt auf Symptome wie Schwindel, Koordinationsstörungen, vermindertes Reaktionsvermögen, verminderte Aufmerksamkeit, Sehstörungen etc. zu achten und im Zweifel auf die aktive Teilnahme am (motorisierten) Straßenverkehr zu verzichten. Denn auch wenn gemäß § 24a StVG das sogenannte Arzneimittel-Privileg gilt und keine Ordnungswidrigkeit vorliegt, sofern die Substanz, die im Blut nachgewiesen worden ist, aus der „bestimmungsgemäßen Einnahme eines für einen konkreten Krankheitsfall verschriebenen Arzneimittels herrührt“, gilt dies im Strafrecht (§§ 315c und 316 StGB) nicht.

Auch Senioren unter Polymedikation ist es generell erlaubt, am Straßenverkehr teilzunehmen, zumal sie in der Regel auf eine jahrelange Fahrpraxis zurückblicken und häufiger gewohnte Fahrstrecken und Fahrtzeiten abseits der Hauptverkehrslast nutzen, wodurch kleinere Fahrauffälligkeiten zunächst kompensiert werden können. Zudem ist der Fahrstil von Senioren häufig ruhiger und rücksichtsvoller, was das Unfallrisiko ebenfalls senkt [29].

Aufgrund von individuellen Faktoren wie Alter, Geschlecht, Vorerkrankungen, Komedikation etc. lässt sich in Bezug auf die Arzneimittel keine pauschale Beurteilung treffen, welches Präparat bei welchem Patienten die Fahrsicherheit beeinträchtigt und falls ja, wie lange. Hier ist eine sorgfältige Auswahl in Absprache zwischen Arzt und Betroffenem vorzunehmen, wobei z. B. auch zu berücksichtigen ist, ob das Kraftfahrzeug (Kfz) immer zu bestimmten Tageszeiten benötigt wird und ob akzeptable, vorübergehende Alternativen zum eigenständigen Führen eines Kfz bestehen.

Allgemein bieten Studienergebnisse Hinweise, welche Arzneimittel für die Anwendung im Alter besser als andere geeignet sind. So konnte zum Beispiel gezeigt werden, dass Zaleplon bei ordnungsgemäßer Einnahme in der Regel keine negativen Auswirkungen auf die Fahrsicherheit am nächsten Morgen hat [13]. Wohingegen Flurazepam insbesondere von betagten Personen in höheren Konzentrationen schlechter vertragen wird [16]. Generell hat sich gezeigt, dass kurz- bis mittellangwirksame Benzodiazepine, die beim Zubettgehen eingenommen werden, weniger im Verdacht stehen, die Fahrsicherheit am nächsten Morgen noch zu beeinträchtigen. Hierbei sollte jedoch trotzdem auf einen ausreichenden Zeitabstand zum Fahrtantritt geachtet werden. Da nicht alle Patienten das Schlafmittel direkt beim Schlafengehen einnehmen, sondern häufig erst, nachdem sie mehrere Stunden wach gelegen haben, sollte insbesondere in diesen Fällen der Zeitabstand bis zur Verkehrsteilnahme nochmals kritisch überprüft werden. Je nach Wirkstoff kann die Wirkung bis zu 11 h (Zopiclon; [14]) oder sogar länger (z. B. Diazepam) anhalten. Benzodiazepine, die tagsüber als Anxiolytikum angewendet werden, sind als ernstes Problem für die Verkehrssicherheit zu betrachten.

Im Fall der Psychopharmaka haben sich die selektiven Serotonin-Wiederaufnahmehemmer als weniger beeinflussend auf die Fahrsicherheit gezeigt als die häufig sedierend wirkenden trizyklischen Antidepressiva.

Bis zum Erreichen einer stabilen Dosierung und dem erfolgten Abklingen von Nebenwirkungen sollte auf eine aktive Teilnahme am (motorisierten) Straßenverkehr verzichtet werden. Insbesondere für Benzodiazepine kann die Fahrsicherheit über mehrere Wochen bis Monate aufgehoben sein und kann auch nach einer Einnahmepause, einem Wechsel des Präparates oder einer Dosisanpassung wieder beeinträchtigt sein.

Dementsprechend ist es erforderlich, dass Personen sich (unabhängig vom Alter) vor Fahrtantritt einer kritischen Selbstprüfung unterziehen. Sofern sie dabei feststellen sollten, dass sie unter kognitiven Defiziten (wie z. B. Konzentrationsstörungen, Verwirrtheitszuständen, herabgesetztem Reaktionsvermögen) und/oder motorischen Defiziten (wie z. B. Gleichgewichtsstörungen, Gang‑/Standunsicherheiten) und/oder Symptomen wie Schwindel, Benommenheit oder Müdigkeit leiden, sollten sie das Autofahren bis zur Symptomfreiheit unterlassen. Darüber hinaus sollten sie diese Beobachtungen mit dem behandelnden Arzt besprechen, sodass ggf. alternative Präparate verordnet werden können oder die Dosis reduziert werden kann. Hierbei sollten auch verschreibungsfreie Arzneimittel, die zusätzlich eingenommen werden, erwähnt werden, weil auch diese zu verkehrsrelevanten Interaktionen mit den ärztlich verordneten Medikamenten führen können.

Generell ist es wichtig, Medikamente nicht eigenständig abzusetzen, wenn Symptome vorliegen, die mit sicherem Autofahren nicht in Einklang zu bringen sind, da Grunderkrankungen unabhängig von der Fahrsicherheit weiterhin adäquat behandelt werden müssen und viele Medikamente nicht abrupt abgesetzt werden dürfen, sondern die Dosierung „ausgeschlichen“ werden muss.

Ein Forschungsverbund, der sich mit verschiedenen Projekten zum Thema Gesundheit im Alter beschäftigt, hat mit der *Priscus-Liste *eine Hilfestellung für Ärzte und Apotheker geschaffen, die sie dabei unterstützt, Medikamente zu identifizieren, die für ältere Menschen potenziell ungeeignet sind, und ihnen alternative Präparate mit einem besseren Nutzen-Risiko-Verhältnis vorschlägt. Des Weiteren finden sich in dieser Liste Dosierungsvorschläge und Überwachungshinweise, um die Medikation von betagten Personen zu optimieren [8]. Als Hilfe für Zuhause bietet sich der Beipackzettel an, der jedem Medikament beiliegen muss. Auch hier sind potenzielle Nebenwirkungen aufgelistet und eine erste Einschätzung zur Fahrsicherheit.

Generell muss hier auch noch einmal auf die ärztliche Aufklärungspflicht hingewiesen werden, da sich viele Menschen nicht der möglichen Nebenwirkungen einer Arzneimitteltherapie und der daraus entstehenden Konsequenzen bewusst sind. Während einer Arzneimitteltherapie sollte grundsätzlich auf den Konsum von Alkohol (oder anderen Drogen) verzichtet werden, insbesondere jedoch bei der aktiven Teilnahme am Straßenverkehr, da die Wirkungen der Arzneimittel durch den Konsum von Alkohol additiv, in der Regel aber überadditiv verstärkt werden.
